# Pulmonary and cardiac drugs: clinically relevant interactions

**DOI:** 10.1007/s00059-019-4834-3

**Published:** 2019-07-11

**Authors:** H. Olschewski, M. Canepa, G. Kovacs

**Affiliations:** 1 Division of Pulmonology, Department of Internal Medicine, Medical University of Graz, Auenbruggerplatz 15, 8036 Graz, Austria; 2 Cardiovascular Unit, Department of Internal Medicine, University of Genova, Genova, Italy

**Keywords:** Heart failure, Chronic obstructive pulmonary disease, Beta-adrenergic blockers, Sympathomimetic drugs, Drug side effects, Herzinsuffizienz, Chronische obstruktive Lungenerkrankung, Betaadrenerge Blocker, Sympathomimetische Medikamente, Medikamentennebenwirkungen

## Abstract

Chronic heart and lung diseases are very common in the elderly population. The combination of chronic heart failure and chronic obstructive pulmonary disease (COPD) is also common and, according to current guidelines, these patients should be treated for both diseases. In patients with heart failure, beta-blockers are very important drugs because their use is associated with significantly improved morbidity and mortality. These beneficial effects were documented in patients with and without COPD, although theoretically there is a risk for bronchoconstriction, particularly with non-beta_1_ selective blockers. In COPD patients, long-acting sympathomimetics (LABA) improve lung function, dyspnea, and quality of life and their combination with a beta-blocker makes sense from a pharmacological and a clinical point of view, because any potential arrhythmogenic effects of the LABA will be ameliorated by the beta-blocker. Inhaled tiotropium, a long-acting muscarinic antagonist (LAMA), has been extensively investigated and no safety concerns were reported in terms of cardiac adverse effects. The same applies for the other approved LAMA preparations and LAMA-LABA combinations. Severe COPD causes air-trapping with increasing pressures in the thorax, leading to limitations in blood return into the thorax from the periphery of the body. This causes a decrease in stroke volume and cardiac index and is associated with dyspnea. All these adverse effects can be ameliorated by potent anti-obstructive therapy as recently shown by means of a LABA-LAMA combination.

## Zusammenfassung

Chronische Herz- und Lungenerkrankungen sind in der älteren Bevölkerung sehr verbreitet. Die Kombination von chronischer Herzinsuffizienz und chronischer obstruktiver Lungenerkrankung (COPD) ist ebenfalls häufig, und diese Patienten sollten, gemäß aktuellen Leitlinien, hinsichtlich beider Erkrankungen behandelt werden. Bei Patienten mit Herzinsuffizienz spielen Betablocker eine sehr wichtige Rolle, weil ihre Anwendung mit einer signifikant verbesserten Morbidität und Mortalität einhergeht. Diese günstigen Auswirkungen wurden sowohl bei Patienten mit als auch bei Patienten ohne COPD dokumentiert, obwohl theoretisch das Risiko einer Bronchokonstriktion besteht, insbesondere bei nicht-beta_1_-selektiven Blockern. Bei COPD-Patienten führen langwirksame Sympathomimetika (LABA) zu einer Verbesserung in Bezug auf Lungenfunktion, Dyspnoe und Lebensqualität, und ihre Kombination mit einem Betablocker ist aus pharmakologischer und klinischer Sicht sinnvoll, da potenzielle arrhythmogene Wirkungen der LABA günstig durch den Betablocker beeinflusst werden. Inhalierbares Tiotropium, ein langwirksamer Muskarinantagonist (LAMA), wurde umfassend untersucht, es bestätigten sich jedoch keine Sicherheitsbedenken hinsichtlich kardialer Nebenwirkungen. Gleiches gilt für die anderen zugelassenen LAMA-Präparate und LAMA-LABA-Kombinationen. Eine schwergradige COPD verursacht eine Lungenüberblähung mit ansteigendem Druck im Thorax, was zur Beeinträchtigung des Blutrückflusses aus der Peripherie des Körpers in den Thorax führt. Dies verursacht eine Abnahme des Schlagvolumens und des Herzindex und geht mit Dyspnoe einher. All diese Nebenwirkungen können durch eine potente antiobstruktive Therapie günstig beeinflusst werden, wie kürzlich mittels einer LABA-LAMA-Kombination nachgewiesen wurde.

Chronic heart disease and lung disease are both very common among people around 70 years of age. The most common single diagnosis in the former group is chronic heart failure and in the latter, chronic obstructive pulmonary disease (COPD). According to the Global Initiative for Chronic Obstructive Lung Disease (GOLD) guidelines, congestive heart failure is among the most important differential diagnoses of COPD [1] and vice versa. In patients with COPD, heart disease is associated with a poor prognosis [2, 3] and in patients with congestive heart failure, COPD is likewise associated with a poor prognosis [4]. Indeed, many patients suffer from both heart failure and COPD.

## Overlap of heart and lung disease

According to the prospective ECLIPSE COPD cohort, nearly 10% of patients in the study had a known diagnosis of chronic heart failure and more than 25% had known “heart trouble” [2]. In a large NHS cohort from the UK with over 31,000 COPD patients and 150,000 matched controls, 17% of the COPD patients suffered from heart failure and 29% from ischemic heart disease [3]. These numbers, although impressive, may still underestimate the problem. In The Netherlands, a cohort of about 400 COPD patients with no known heart disease underwent thorough multidisciplinary examinations in a specialized clinic. It was found that, based on echocardiography, 10% suffered from heart failure with reduced ejection fraction (HFrEF) and another 10% from heart failure with preserved ejection fraction (HFpEF; [5]). By far the highest rates of pathologic findings were found in patients over 75 years of age, both in males (mostly HFrEF) and females (mostly HFpEF). This suggests that at least 30% of COPD patients suffer from concomitant heart failure. And vice versa, when we look into large congestive heart failure cohorts, around 30% also suffer from COPD [4, 6, 7]. This means that the elderly population in developed countries presents with a broad overlap of congestive heart failure and COPD.

There have been several approaches to explain these strong associations, ranging from shared risk factors like smoking, to shared pathologic mechanisms like systemic inflammation, to shared genetic factors. The most comprehensive concept, however, was delivered by an American Thoracic Society (ATS)/European Respiratory Society (ERS) Task Force report, interpreting COPD “as the pulmonary component of a chronic multimorbidity.” This concept explains why COPD is associated with many common risk factors, such as smoking, pollution, ageing, inactivity, and diet [8]. This also implies that COPD and congestive heart failure are not only very common in the adult population but also often combined in a single subject, i.e., COPD can be considered as a risk factor for congestive heart failure and vice versa. In addition, the Copenhagen City Heart Study showed that COPD also represents a strong risk factor for atrial fibrillation and a moderate risk factor for systemic arterial hypertension [9]. This was confirmed in the most recent large NHS cohort [3].

## Beta-adrenergic receptor: important pharmacologic target for congestive heart failure and COPD

In congestive heart failure patients, beta-blockers are one of the most important therapeutic options because they significantly improve morbidity and mortality [10]. By contrast, COPD patients profit from beta-sympathomimetics, drugs that have been shown to improve lung function, dyspnea, and quality of life [11]. This may sound like a contradiction: How should we then treat patients who suffer from both COPD and congestive heart failure?

For COPD patients, the GOLD guidelines [11] and the ERS recommendations [12] provide a clear statement: “Comorbidities should be treated as if the patient did not have COPD.”

In cases of obstructive lung diseases, beta-sympathomimetics (LABA) are approved for COPD and asthma and they are recommended by the current guidelines for COPD [13] and asthma [14] although there is no evidence for a beneficial effect on mortality. Most of the evidence has been generated for combination treatments: for asthma with inhaled corticosteroids (ICS-LABA), and for COPD with long-acting muscarinic antagonists (LAMA-LABA). According to the GOLD and the ERS guidelines, these medications can be prescribed for symptomatic COPD patients even if significant heart disease is present.

In cases of congestive heart failure as a comorbidity of COPD, the GOLD/ERS recommendation [11, 12] means that patients with an indication for a beta-blocker should indeed receive a beta-blocker. According to the current guidelines for acute and chronic heart failure [10] and the Global Initiative for Asthma (GINA) guidelines for asthma [14], even asthma is not an absolute contraindication for beta-blockers, despite reports of adverse effects from the 1980s and 1990s with mostly unselective beta-blockers at high initial doses in patients with severe asthma. For patients with ischemic heart disease [15, 16] and with heart failure [17], beta-blocker use was associated with a significant beneficial effect on mortality. This was not only true for the whole study population but particularly for the subgroup of patients with concomitant COPD. In addition, beta-blockers are important medications for controlling atrial fibrillation and systemic hypertension.

## Receptor specificity and route of application

### Beta-blockers

**Fig. 1:**
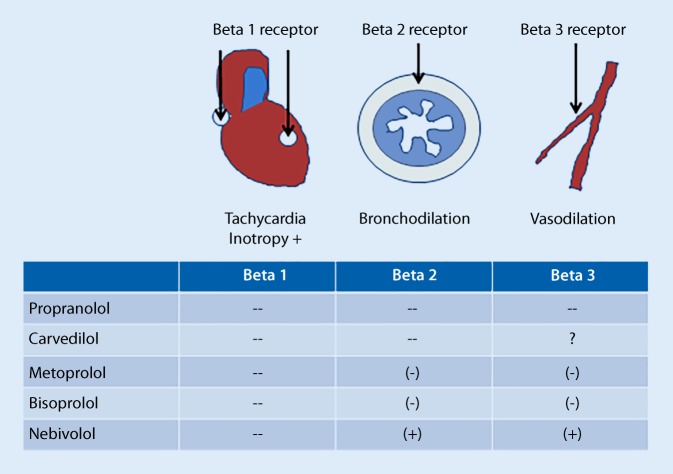
Beta receptors, their main function in cardiovascular and pulmonary physiology, and the most important beta-blockers for clinical use

Cardiovascular diseases are the most frequent and important comorbidities of COPD and include ischemic heart disease, congestive heart failure, and atrial fibrillation. For these diseases there are clear indications for beta-blockers. In COPD patients, beta_1_ selective beta-blockers are considered to be advantageous, although there are also data for the unselective beta_1_/beta_2_ adrenergic blocker carvedilol suggesting a good tolerability in COPD patients [18]. Still, a potential problem is the large overlap between COPD and asthma. Up to 30% of COPD patients may suffer from asthma as a comorbidity [19] and asthma patients may develop adverse effects from nonselective beta-blockers. If the beta_1_/beta_2_ effect of propranolol was 1, bisoprolol would have a ratio of 103 and nebivolol of 321 [20]. Nebivolol is a substance that has strong beta_1_-blocking properties, virtually no effect on the beta_2_ receptor and at the same time is a beta_3_ receptor agonist (Fig. 1). This profile might be particularly advantageous for patients with heart disease and COPD with an asthmatic component and/or pulmonary arterial vasoconstriction. However, because there was no significant beneficial effect of nebivolol on mortality in a recent study with Asian patients [21], nebivolol was not recommended by all guidelines for chronic heart failure.

### Beta-sympathomimetics

Beta-sympathomimetics improve lung function and dyspnea by means of their bronchodilating properties via activation of the beta_2_ receptor of the bronchial smooth muscle cells (Fig. 1). Principally they can be delivered systemically or via the inhaled route. Unfortunately, none of the available beta_2_ agonists is highly selective for the beta_2_ receptor and they all may cause beta_1_ agonistic effects like tachycardia and increased oxygen demand of the heart. Therefore, systemic application, which is associated with many more cardiac side effects than inhaled application, is not recommended. In addition, the approved substances have different pharmacologic profiles. The short-acting beta_2_ agonists (SABA), like salbutamol and fenoterol, were the first available substances, followed by long-acting beta_2_ agonists (LABA) like salmeterol for twice daily use and ultra-long-acting substances like indacaterol, olodaterol, and vilanterol for once daily use. As a rule, immediate onset of bronchodilatation is associated with a short duration, and a slow onset with a long duration of the drug. However, there is one exception to the rule: Formoterol has both a rapid onset and a long duration of bronchodilation. This is why the GINA guidelines recommend only formoterol as an on-demand combination drug in Step 1 and Steps 3–5 of asthma therapy [14]. Although there is no head-to-head comparison, LABA are considered superior to SABA for chronic stable asthma and COPD disease, because they are less prone to causing tachycardia and other cardiac side effects.

## Advantages of combined beta-blocker plus beta-sympathomimetic therapy

When the patient is treated with an unselective beta-blocker, a concomitant LABA therapy may ameliorate any bronchoconstrictive effects. And vice versa, any adverse effects of the LABA on the heart will be ameliorated by the beta-blocker. When the patient is treated with a beta_1_ selective beta-blocker, even a high-dose beta-blocker will not cause bronchoconstriction, because the beta receptors are stimulated by the LABA. And vice versa, as the LABA is delivered via the inhaled route, it will not cause major cardiac side effects and any minor side effects will be ameliorated by the beta-blocker.

In summary there is an excellent rationale for treating patients suffering from heart failure and COPD with both a beta_1_ selective beta-blocker and an inhaled long-acting or ultra-long-acting beta_2_ agonist.

## Potential interactions between cardiac medications and COPD

Aspirin is a mainstay of secondary prevention in ischemic heart disease. In addition, it has a significant preventive role for recurrent venous thromboembolism [22]. Aspirin increases the risk of bleeding and this may affect COPD patients more than other patients owing to coughing, osteoporosis, and fragile small vessels. In addition, COPD may be associated with asthma and among these patients some will be sensitive to aspirin and other nonsteroidal anti-inflammatory drugs (NSAIDs). This special asthma phenotype is often associated with “late-onset asthma” and nasal polyposis. However, since the aspirin for ischemic diseases is taken on a daily basis, there will be a desensitization of the asthma mechanism and adverse effects on the airways are very unlikely.

Oral anticoagulants are often indicated in ischemic and congestive heart disease and in atrial fibrillation. Patients with COPD may be more prone to bleeding complications than patients without COPD. This is very obvious in the skin but also affects the abdominal muscles and other organs. Severe coughing in combination with osteoporosis represents a strong risk factor for rib and vertebral fractures. In such cases, anticoagulation may cause significant complications.

Amiodarone is still the most potent anti-arrhythmic drug and is used for ventricular and supraventricular arrhythmia. Unfortunately, there is a dose-dependent toxicity that can affect many organs. In the lung it causes alveolitis, which is associated with worsening of pulmonary gas exchange and severe hypoxemia. Because of the extremely long half-life of amiodarone, it may take many weeks after cessation of amiodarone treatment until the alveolitis improves. The risk of life-threatening complications of alveolitis is increased in subjects with pre-existing lung disease like COPD.

Angiotensin-converting enzyme (ACE) inhibitors (ACEi) remain important drugs for the treatment of congestive heart failure. However, they may frequently cause chronic cough and rarely they may cause angioedema. In COPD patients, cough is common and ACEi-induced cough may be mistaken for COPD or asthma exacerbations. This may explain why in hospitalized patients with concomitant COPD, compared to those without COPD, ACEi were less frequently applied [23].

## Potential interactions between COPD medication and heart disease

Long-acting muscarinic antagonists (LAMA) have been one of the most important therapy options for COPD since the first drug of this class was approved in 2008. Since then, four substances—tiotropium, glycopyrronium, aclidinium bromide and umeclidinium—have been approved for inhaled use in COPD. Before 2008, only ipratropium, a short-acting muscarinic antagonist (SAMA), was available for inhaled use. A meta-analysis suggested significant adverse cardiovascular outcomes for studies comparing ipratropium with placebo and for all studies where either ipratropium of tiotropium was compared with placebo [24]. However, a randomized controlled study with tiotropium, delivered by the HandiHaler device, showed significant beneficial effects of tiotropium on adverse cardiac events [25]. After introduction of the Respimat inhaler for tiotropium, there were again safety concerns because of reports of adverse cardiovascular outcomes as compared with the tiotropium HandiHaler. However, a large prospective randomized controlled study with the Respimat device showed no evidence for increased cardiovascular adverse effects [26]. In conclusion, LAMA are considered safe in cardiac patients, although high-quality evidence for safety from large databases is only available for tiotropium.

Theophylline (dimethylxanthine) has been used for airway obstruction since 1922. Xanthines are known to inhibit several phosphodiesterases (PDE-3, 4, 5, 7, 9) that are present in bronchial smooth muscle cells and inflammatory cells. This has bronchodilatory and anti-inflammatory effects [27]. However, xanthines also inhibit adenosine receptors (AR-A1, A2A, A2B, A3), which may cause cardiac side effects. Among the different xanthine drugs, doxophylline has strong PDE inhibitory effects and modest adenosine receptor effects, which translates into clinical superiority over other xanthines [28]. According to current guidelines, xanthines are not considered as first-line medications for COPD or asthma. However, in patients who do not tolerate beta agonists, they may still have a place, particularly in younger adults with no cardiac disease. A newly developed PDE inhibitor (roflumilast) is recommended for patients with severe COPD with productive cough who are not underweight [13]. The drug often causes diarrhea, nausea, and weight loss. This profile might be particularly advantageous to patients at risk for chronic heart disease due to over-nutrition.

Oral corticosteroids (OCS) should be avoided in the chronic treatment of COPD and asthma; however, some patients respond with frequent exacerbations after cessation of OCS. Interleukin-5 inhibitors have been approved to avoid or reduce OCS but only in asthma and not in COPD. During acute exacerbations of COPD and asthma, OCS with 0.5 mg/kg prednisolone for 5 days [29] is recommended because this therapy leads to relief of obstruction and symptoms and shortens the hospitalization [11]. Unfortunately, OCS are associated with a multitude of adverse events among which venous thromboembolism is of special interest for COPD patients because it may mimic COPD exacerbation [30] and is more difficult to diagnose than in patients without structural lung disease. It is also of interest for chronic heart failure because these patients per se have a significantly increased risk for venous thromboembolism.

## Chronic cor pulmonale

Pulmonary disease may affect the heart, and the most obvious consequence of chronic lung disease on the heart is chronic cor pulmonale. This observation was made by ancient pathologists and defined as the presence of a dilated right ventricle in a patient without obvious left heart disease. However, the MESA COPD study, by means of systematic magnetic resonance imaging (MRI) and pulmonary function test (PFT), found that the right ventricular volume in stable COPD patients was smaller by about 8 ml compared with healthy matched controls [31]. There was a highly significant correlation between the degree of airway obstruction and the reduction of the right ventricular volume, and there was a highly significant correlation between the amount of emphysema and the reduction in right ventricular volume. This led to the hypothesis that the positive intrathoracic pressure in obstructive lung disease prevents blood flow into the thorax and causes underfilling of the heart.

A recent interventional study demonstrated that this hypothesis was true and that the mechanism was reversible. By means of a potent antiobstructive therapy, using an inhaled LABA/LAMA combination for 2 weeks in patients with severe COPD with no cardiac disease, the right ventricular volume increased by about 7 ml, along with a significant increase in stroke volume and cardiac output [32]. These changes were due to a significant reduction in the pulmonary residual volume and altogether resulted in a highly significant beneficial effect on dyspnea and quality of life. This suggests that air trapping, due to obstructive lung disease causes intrathoracic pressure increase, resulting in cardiac underfilling and hemodynamic stress and that these pathologic findings can be reversed by potent bronchodilatory therapy. However, such effects may be much less impressive if the patients suffer from some degree of left heart disease [33].

## Conclusion

In conclusion, the combination of chronic heart and lung diseases is very common in the elderly population. Patients should be treated for both diseases. Beta-blockers are very important drugs because their use is associated with significantly improved morbidity and mortality, and their combination with inhaled long-acting sympathomimetics makes sense from a pharmacological and a clinical point of view. There are numerous potential effects of heart medications on the diseased lung and of lung medications on the diseased heart. However, in most instances, such medication is justified, just as in cases where the other disease is not present.
